# Assessment of mastoid emissary foramen morphology: a multidetector computed tomography study

**DOI:** 10.1007/s11282-025-00808-3

**Published:** 2025-02-25

**Authors:** Ahmet Faruk Ertürk, Gürkan Ünsal, Sevde Göksel, Elif Çelebi, Hamit Tunç, Maria Maddalena Marrapodi, Marco Cicciù, Giuseppe Minervini

**Affiliations:** 1 Department of Dentomaxillofacial Radiology, Biruni University, Istanbul, Turkey; 2 Schulich School of Medicine & Dentistry, Western University, London, ON Canada; 3 Tepebaşı Oral and Dental Health Hospital, Ankara, Turkey; 4 Department of Oral and Maxillofacial Radiology, Bahçeşehir University, Istanbul, Turkey; 5 Department of Pedodontics, Burdur Mehmet Akif Ersoy University, Burdur, Turkey; 6 Department of Woman, Child and General and Specialist Surgery, University of Campania “Luigi Vanvitelli”, 80121 Naples, Italy; 7 Department of Biomedical and Surgical and Biomedical Sciences, Catania University, 95123 Catania, Italy; 8 Saveetha Dental College and Hospitals, Saveetha Institute of Medical and Technical Sciences (SIMATS), Saveetha University, Chennai, 600077 India; 9 Multidisciplinary Department of Medical-Surgical and Dental Specialties, University of Campania Luigi Vanvitelli, 80138 Naples, Italy

**Keywords:** Mastoid emissary foramen, Mastoid emissary vein, Mastoid emissary canal, Multidetector computed tomography

## Abstract

**Objectives:**

This study aimed to assess the occurrence and morphological features of the mastoid emissary foramen (MEF) using multidetector computed tomography (MDCT) images. The analysis highlights the clinical significance of these structures and their implications for surgical procedures.

**Methods:**

A total of 357 patients were evaluated using MDCT in bone window mode with a high-resolution technique (1 mm). The presence, number, and mean diameter of the MEFs were recorded. Statistical analyses compared data between both sides and sexes.

**Results:**

714 sides from 357 patients (177 male, 180 female) were analyzed. The patients’ ages ranged from 7 to 83 years, with a mean age of 25.6. MEFs were found in 329 patients, representing 92.15% of the total. The diameters of the MEFs ranged from 0.6 mm to 5.0 mm on the right side (mean 1.80 mm) and from 0.6 mm to 4.4 mm on the left side (mean 1.96 mm). Up to 3 MEFs were identified on the right side, and a maximum of 6 on the left. No significant differences in MEF presence were observed between sexes or between the left and right sides (*p* > 0.05).

**Conclusion:**

This study reveals a high prevalence and notable anatomical variations in the MEF, with MEFs larger than previously reported. At least one MEF was detected in 92.15% of cases, emphasizing the importance of comprehensive preoperative evaluation.

## Introduction

Emissary veins are vessels that connect extracranial veins to intracranial venous sinuses, passing through emissary foramina in the skull. Since these veins lack valves, they allow bidirectional blood flow, helping maintain stable pressure within the venous sinuses. The mastoid emissary vein (MEV) specifically connects the posterior auricular vein to the sigmoid venous sinus. It originates from the sigmoid sinus, travels through the mastoid emissary canal (MEC), and exits via the mastoid emissary foramen (MEF), located behind the mastoid process, sometimes near the occipitomastoid suture. The MEV also passes through a meningeal branch of the occipital artery within the MEC, which is positioned near the mastoid process of the temporal bone [1–3]. The MEC is a small canal, typically 0.5 to 1 cm long, with a thin bony wall and a lumen lined with mucosal tissue [4–6].

In healthy individuals, blood flow within the MEV is typically slow, moving from the intracranial venous system to the extracranial veins. However, when intracranial pressure rises, the MEV carries more blood, helping to lower intracranial pressure. As a key pathway for cerebral venous drainage, the MEV can enlarge in cases of intracranial hypertension, hypoplasia, or the absence of internal jugular veins. This enlargement can lead to increased blood flow in the region, potentially raising the risk of hemorrhage during surgery. Injury to the MEV may result in serious complications such as bleeding, sigmoid sinus thrombosis, cerebellar ischemia, and, in extreme cases, death. Therefore, understanding the MEV’s trajectory, size, location, and number is critical for avoiding intraoperative bleeding and postoperative complications [1, 7]. Additionally, the increased diameter of the MEV in patients with chronic otitis media is a crucial factor to consider in surgical procedures [8].

Anatomical and morphological studies of the MEF, MEV, and MEC have been conducted, with most of the research based on cadaver examinations. However, only a few radiological studies have focused on the morphology of these structures. Radiological imaging plays a crucial role in both surgical planning and postoperative evaluation, offering valuable insights for clinicians. By mapping the pathways of emissary foramina and veins, radiologists can help predict potential complications and improve clinical outcomes. Despite its significance, the importance of the MEV has been relatively overlooked, though it can lead to unexpected complications during surgery, such as bleeding, and in rare cases, patient death [4]. Understanding the precise location and variations of the MEC is vital to reducing surgical complications in this region. This retrospective study aimed to evaluate the prevalence and perform a morphometric analysis of the MEF using multidetector computed tomography (MDCT).

## Materials and methods

### Ethics statement

The study protocol was approved by the Ethics Committee of Mehmet Akif Ersoy University (Meeting No: 2024/05, Decision No: GO 2024/307). The study was conducted in compliance with the principles of the 1964 Helsinki Declaration and its later amendments. Informed consent was obtained from all participants prior to their inclusion in the study.

### EQUATOR guideline

The CRIS Guidelines of the EQUATOR Network are followed in this study. “Sample size calculation, meaningful difference between groups, sample preparation and handling, allocation sequence, randomization, and blinding statistical analysis” are the requirements that the present research satisfies.

### Eligibility

Volunteers who will form the sample are individuals who have had cranial MDCT imaging for any reason, and the inclusion criterion for the research is having undergone cranial MDCT imaging. Exclusion criteria were presence or history of bone surgery, infectious diseases such as chronic otitis media, tumor and trauma affecting posterior cranial fossa, neck or temporal region.

### Sample size

The calculated sample size required to estimate the prevalence of MEF on MDCT images with a 95% confidence level and a margin of error of ± 5% was approximately 180 participants. A sample size of 357 patients (177 male, 180 female) was achieved from the archive, thought to be sufficient to meet the objectives of the study while balancing statistical power and practical feasibility.

### Morphology and morphometry

Two researchers, each with 6 years of experience in Oral and Maxillofacial Radiology, independently analyzed the MDCT images retrospectively. Before conducting the analysis, both researchers underwent training to standardize and ensure consistency in measurement techniques. Interobserver correlation coefficients were calculated to assess the consensus between the two evaluators. All images were acquired using the same MDCT device under the bone window mode.

The researchers evaluated the presence and number of MEF and compared differences between sexes as well as between the right and left sides. For morphometric analysis, the diameter of the MEF was measured using MultiPlanar Reconstruction (MPR) views in the OnDemand 3D Viewer (OnDemand 3D^™^ by Cybermed, California, USA), displayed on an Advantech KT R240FEE Medical LCD Monitor (Advantech by Kostec, Gangwon, South Korea) to enhance image quality and accuracy.

The evaluators were able to freely manipulate the volume in three orthogonal planes (axial, coronal, and sagittal) to precisely locate the MEF and identify the optimal section for measurement. The largest diameter of each MEF was measured in the plane where it appeared most clearly, typically in the axial view. If the MEF was not perfectly round, both the largest and smallest diameters were recorded using perpendicular axes (horizontal and vertical). For consistency, the largest diameter was used in statistical analyses.

The evaluators measured the outermost points of the MEF, ensuring the measurements included the entire span of the foramen from edge to edge. The volume was not adjusted, but evaluators could rotate and navigate through the reconstructed planes to ensure accurate placement of measurement points at the outer edges of the MEF.

**Fig. 1:**
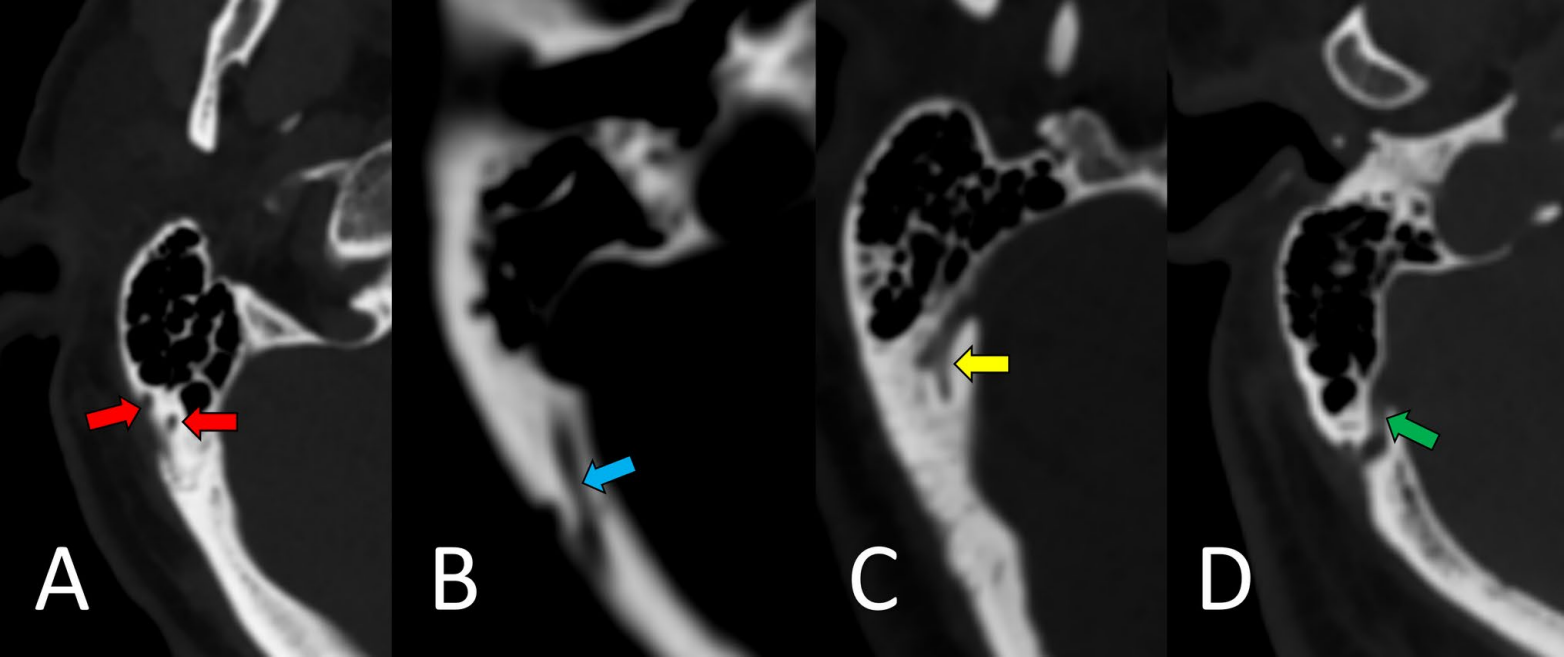
Axial slice CT images demonstrate a variety of morphologies of MECs, **A** two evident MEFs (red arrows), **B** a long MEC (blue arrow), **C** a single MEC that divides into two MECS with a bifurcation (yellow arrow), **D** and a MEC with a large diameter (green arrow), showing examples of diverse anatomical features

All measurements were conducted twice by each evaluator to ensure reliability and any discrepancies were resolved through discussion and reanalysis. The mean diameters of the MEFs and MECs were then calculated (Fig. 1)**.**

### MDCT device

The imaging system used for this retrospective study was the GE Discovery CT750 HD 64 sectioned MDCT device (General Electric Healthcare, Chicago, IL, USA). The MDCT device is powered by a 100 kW generator and employs a Performix™ HD 8Mhu X-ray tube. It offers a wide capacity range, from 10 to 835 mA, with 5 mA increments, and provides selectable kVp options of 80, 100, 120, and 140 kVp. The MDCT device uses Exclusive V-Res^™^ Detector technology, which enables a coverage of 40 mm for each rotation. The gantry of the device allows rotation times between 0.35 s to 1 s, utilizing the VariSpeed feature. The image chain comprises ASiR (Adaptive Statistical Iterative Reconstruction) radiation dose reduction technology and has a 64-channel configuration, enabling 64 slices per rotation. A complete scan takes 0.35 s, producing thin slice images with a thickness of 0.625 mm.

### Statistical methods

Statistical analysis was performed using IBM SPSS Statistics for Windows, Version 21.0 (IBM Corp., Armonk, NY, USA). The Analysis of Variance Test (ANOVA) was employed for the comparison of variables. Statistical significance was set at *p* < 0.05. In the correlation analyses, the relationship between the right and left MEF values and age was assessed using the Spearman correlation test.

## Results

In our analysis of 357 patients (714 sides), aged between 7 and 83 years with a mean age of 25.6, we identified a total of 978 MEFs, resulting in an overall prevalence of 92.15%. MEFs were present on the right side in 322 patients and on the left side in 323 patients, with no significant difference in prevalence between the two sides (*p* > 0.05). On average, patients with MEFs had approximately 2.97 foramina per individual, with the maximum number of MEFs observed being 3 on the right side and 6 on the left side.

The diameters of the MEFs varied across patients. On the right side, diameters ranged from 0.6 mm to 5.0 mm, with a mean diameter of 1.80 mm. On the left side, diameters ranged from 0.6 mm to 4.4 mm, with a mean diameter of 1.96 mm. A subset of patients (4 out of 357) had two MEFs with diameters larger than 3.5 mm, which is a clinically relevant threshold due to the potential for surgical complications. Additionally, two patients had a single MEF exceeding 3.5 mm.

Statistical analysis showed no significant differences in MEF prevalence between males and females or between the left and right sides (*p* > 0.05). Similarly, no significant differences were found in MEF frequency between sexes for either the left or right sides (*p* > 0.05) (Table 1). However, a significant difference was observed between bilateral and unilateral MEFs, which may be attributed to the disparity in the number of foramina between the two groups (*p* < 0.05) (Table 2).

**Table 1:** Comparison of the mean diameter of MEFs between sexes

	mean ± sd [min–max]		
Sex (n)	Male (165)	Female (164)	p*
Right Side	2.22 ± 0.80 [0–3.4]	2.17 ± 0.82 [0–5]	0.597
Left Side	2.18 ± 0.80 [0–3.4]	2.29 ± 0.78 [0.6–4.1]	0.205

^*^ANOVA Test

**Table 2:** Evaluation of the mean diameter of MEFs according to laterality

	mean ± sd [min–max]		
Presence (n)	Unilateral (13)	Bilateral (316)	p*
Right Side	1.16 ± 1.32 [0–2.8]	2.24 ± 0.75 [0.6–5]	0.00
Left Side	0.70 ± 0.68 [0–1.5]	2.30 ± 0.73 [0.6–4.1]	0.00

^*^ANOVA Test

**Fig. 2:**
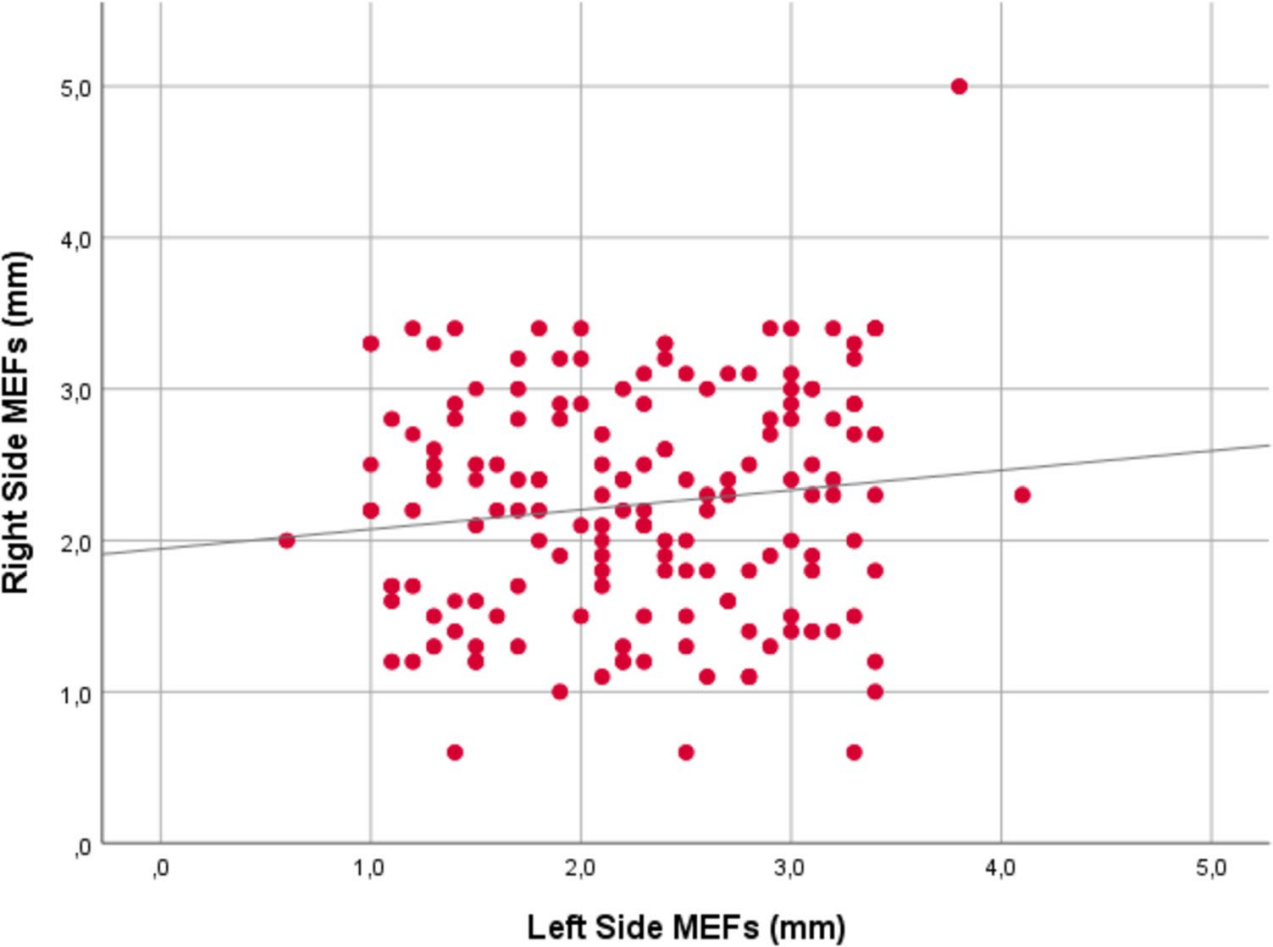
Scatter plot showing the correlation between the diameters (mm) of variations on the right and left sides

Further analysis revealed that both unilateral and bilateral MEFs had larger diameters on the right side compared to the left side. A weak positive correlation was noted between the diameters of MEFs on the right and left sides (*p* = 0.02, *r* = 0.128), as illustrated in Fig. 2. No statistically significant correlation was observed between age and right-side MEF diameters (*p* = 0.458). Similarly, no significant correlation was detected between age and left-side MEFs (*p* = 0.549).

Lastly, the interobserver correlation for MEF diameter measurements was 0.912, indicating strong agreement between examiners.

## Discussion

The morphometric characteristics of the MEC have been widely studied, focusing on its prevalence, size, and clinical significance. While previous studies have reported the prevalence of MEC to range from 88.1% to 91.7% [3, 6, 9], it has been observed more frequently on the right side [3–5]. In our study, we observed at least one MEF in 92.15% of cases, which represents the highest prevalence reported in the literature. Additionally, we identified a unique case with six MEFs on the left side, which surpasses previous reports of up to four MEFs. Louis et al. (2009) classified the number of MEFs from 0 to 4 into five types (Type I: single, Type II: double, Type III: triple, Type IV: quadruple, and Type V: absent) [10]. In our study, most cases fell between Types I-III, with one case classified as Type IV, and the exceptional case with six MEFs remaining unclassified. Yurdabakan et al. (2023) reported that a single MEF was observed in 69.4% of cases, double in 10.3%, and triple in 1.6%, with no MEF present in 18.6% of cases. Quadruple MEFs were rare, appearing in only one case on the right side [11].

The clinical importance of accurately identifying and examining the MEF preoperatively cannot be overstated, given the significant variations in size, location, and course [1, 4, 6]. Enlarged MEFs have been associated with symptoms such as tinnitus [4, 12, 13], compressive scalp masses [14], thrombophlebitis [15], and facial swelling [16]. Also, a study by Ozen and Sahin [8] reported significantly larger MEF diameters in patients with chronic otitis media compared to controls, which is important for surgical planning.

In cases where the morphometric structure of the MEC is poorly understood, particularly when its diameter exceeds 3.5 mm, unexpected complications can arise during procedures such as mastoidectomy, epitympanectomy, and suboccipital craniotomy. These complications may include epidural and subdural hematomas resulting from unexpected bleeding [1]. In our study, six patients had at least one MEF with a diameter greater than 3.5 mm, a clinically significant threshold. According to the literature, the largest reported MEF diameters range from 4.5 mm to 8.9 mm [4, 10, 11, 17, 18]. In our sample, two patients had MEFs with diameters of 5 mm. Generally, an MEF with a diameter of 3.5 mm or greater is considered large [19]. Furthermore, the MEV canal is classified as prominent if its width exceeds 1 mm [20].

The variation in the number, size, and diameters of MEFs between individuals, and even between the right and left sides of the same patient, showed considerable standard deviations in our study. However, no statistical significance was found for these variations. Previous studies have reported varying average diameters of MEFs. Louis et al. (2009) evaluated cadaver heads and dried human skulls, reporting a mean MEF diameter of 3.5 mm, with a range of 1.1 to 5.6 mm [10]. Yurdabakan et al. (2023) analyzed 472 Cone-beam computed tomography (CBCT) images and found mean diameters of 3.39 ± 1.48 mm for the MEF and 2.05 ± 1.06 mm for the MEC, with males having larger mean diameters than females [11]. Similarly, Temiz et al. (2023) reported mean diameters of 2.4 ± 0.9 mm for the MEF and 2.1 ± 0.8 mm for the MEC, again noting significantly larger diameters in males [6]. In our study, we found a mean MEF diameter of 2.22 ± 0.80 mm on the right side in males and 2.17 ± 0.82 mm in females. On the left side, the mean MEF diameter was 2.18 ± 0.80 mm in males and 2.29 ± 0.78 mm in females. However, we did not observe significant differences in MEF diameters based on sex.

The morphology of the MEC has been explored using various imaging modalities, including MDCT [2, 7, 8], CBCT [6, 11], and magnetic resonance imaging [5, 21]. A study by de Oliveira-Neto et al. (2021) proposed a new classification system for MEF and MEV based on their three-dimensional morphology using MDCT [7]. The importance of using high-resolution imaging techniques such as MDCT or CBCT, prior to surgery has been highlighted due to the clinical implications of MEC variations and potential complications during surgical procedures [3, 6]. Roser et al. (2014) compared helical CT scans reconstructed using standard techniques (4.5-mm slices) with high-resolution techniques (1 mm) and found that the latter significantly improved the detection rate of emissary veins [22]. This difference in imaging techniques may explain some discrepancies between studies. In our study, the use of MDCT with a 1 mm voxel size enhanced the visualization of smaller anatomical structures. For example, Zhou et al. (2023) reported detecting MEFs in only 49.0% of patients using conventional CT scans, which had lower sensitivity [23]. In contrast, the high-resolution imaging in our study allowed us to identify a patient with six MEFs, surpassing previously reported maximums and highlighting the value of enhanced imaging techniques for detecting anatomical variations.

The strength of our study lies in the use of MDCT with a 1 mm voxel size and the employment of two experienced radiologists for measurements, which ensured reliability. However, as this was a retrospective observational study, it did not allow for the analysis of clinical outcomes related to the morphological and dimensional variations of MEFs. Further research is needed to explore recently proposed morphological classifications and to examine the relationship between MEF morphology and surgical complications.

## Conclusions

In conclusion, this study provides valuable insights into the prevalence and morphometric characteristics of the MEF using MDCT. The findings revealed that MEF was present in 92.15% of the cases, with a mean of approximately 2.97 foramen per patient. The diameter of the MEF varied between the right and left sides, with some cases showing up to six foramina on the left side, which surpasses previously reported values. No significant difference in MEF prevalence was observed between sexes or between the left and right sides. The high prevalence and substantial anatomical variation of the MEF emphasize the importance of comprehensive preoperative evaluation to minimize the risk of complications during surgery. Continued research using advanced imaging techniques will help refine surgical approaches and improve patient outcomes.

## Data Availability

The data that support the findings of this study are available from the corresponding author, AFE, upon reasonable request.
